# Combination Therapy Using Monoclonal Antibodies against Respiratory Syncytial Virus (RSV) G Glycoprotein Protects from RSV Disease in BALB/c Mice

**DOI:** 10.1371/journal.pone.0051485

**Published:** 2012-12-27

**Authors:** Hayat Caidi, Jennifer L. Harcourt, Ralph A. Tripp, Larry J. Anderson, Lia M. Haynes

**Affiliations:** 1 National Center for Immunization and Respiratory Diseases, Division of Viral Diseases, Gastroenteritis and Respiratory Viruses Laboratory Branch, Centers for Disease Control and Prevention (CDC), Atlanta, Georgia, United States of America; 2 College of Veterinary Medicine, Department of Infectious Diseases, University of Georgia, Athens, Georgia, United States of America; 3 Division of Pediatric Infectious Diseases, Emory Children’s Center, Atlanta, Georgia, United States of America; University of Iowa, United States of America

## Abstract

Therapeutic options to control respiratory syncytial virus (RSV) are limited, thus development of new therapeutics is high priority. Previous studies with a monoclonal antibody (mAb) reactive to an epitope proximal to the central conserved region (CCR) of RSV G protein (mAb 131-2G) showed therapeutic efficacy for reducing pulmonary inflammation RSV infection in BALB/c mice. Here, we show a protective effect in RSV-infected mice therapeutically treated with a mAb (130-6D) reactive to an epitope within the CCR of G protein, while treatment with a mAb specific for a carboxyl G protein epitope had no effect. Combined treatment with mAbs 130-6D and 131-2G significantly decreased RSV-associated pulmonary inflammation compared to either antibody alone. The results suggest that anti-RSV G protein mAbs that react at or near the CCR and can block RSV G protein-mediated activities are effective at preventing RSV disease and may be an effective strategy for RSV therapeutic treatment.

## Introduction

Respiratory syncytial virus (RSV) is an important cause of acute lower respiratory tract in infants and the elderly [1], [2] resulting in considerable morbidity and a substantial number of hospitalizations in the United States each year [3], [4]. Unfortunately, there is no licensed RSV vaccine and treatments are limited to ribavirin which is woefully inadequate. [5], [6], [7] Ribavirin is licensed for treatment of severe RSV infection but has limited efficacy and is seldom used except for treatment of RSV infection in immune compromised patients [8]. An explanation for the ineffectiveness of ribavirin and other anti-virals is that the virus-induced inflammatory response generated during infection is an important contributor to disease pathogenesis and facets persists after virus replication has ended [9], [10]. It is important to note that while prophylaxis with palivizumab, a humanized IgG monoclonal antibody (mAb) directed against the F protein of RSV, has demonstrated effectiveness in reducing hospitalization; it is not recommended in treating RSV once infection is established [9].

Several studies have shown that the RSV attachment (G) protein has a substantial role in inducing and modulating the host immune response to infection [11], [12], [13], [14], [15]. RSV G protein is approximately 50% conserved among predominant RSV strains, but contains two conserved regions: the cytoplasmic/transmembrane region (amino acids a.a 1 to 66) and a central conserved region (CCR) from a.a 148–198 [16], [17]. Within the central conserved region of RSV G protein is a CX3C chemokine motif between a.a 182 to 186 that functionally mimics the CX3C chemokine fractalkine (FKN) [18]. Through this motif, the RSV G protein binds to the fractalkine receptor, CX3CR1, and facilitates virus infection. RSV G CX3C-CX3CR1 interaction is associated with altered pulmonary leukocyte trafficking, altered Th1-type cytokine and C–C/CXC chemokine expression and increased pulmonary substance P levels [11], [14].Intriguingly, a variation in the CX3CR1 gene has been associated with increased risk for severe RSV bronchiolitis in children hospitalized for bronchiolitis, supporting the importance of G protein CX3C-CX3CR1 interaction in disease pathogenesis [19]. Blocking RSV G protein binding to CX3CR1 using an anti-RSV G monoclonal antibody (mAb 131-2G) that reacts proximal to the central conserved region (amino acids 1–173) inhibited RSV G protein-induced leukocyte migration in vitro [18], and reduced pulmonary inflammation in RSV-infected mice given early therapeutic, or prophylactic administration of mAb 131-2G [21], [22], [23].

These findings led to the hypothesis that anti-RSV G protein mAbs that recognize different epitopes near to or within the CX3C region of G protein may act to block CX3C-CX3CR1 related functions, and if used in combination, would act to enhance the efficacy of antibody treatment and reduce RSV-associated disease. In this study, monoclonal antibodies that react to an epitope in the central conserved region that blocks RSV G binding to CX3CR1 (130-6D), or react to an epitope outside the central conserved region and is poor at blocking RSV G binding to CX3CR1 (mAb 232-1F), were evaluated for their therapeutic efficacy. The results show that mAb 130-6D reduces inflammatory parameters associated with pulmonary disease in RSV-infected mice, and blocks RSV G protein induced leukocyte migration. In addition, the results show that the protective efficacy is increased when administered in combination with mAbs that recognize different epitopes near to or within the CX3C region of G protein (131-2G), an effect that reduces bronchoalveolar lavage (BAL) cell infiltration, and viral gene expression and interferon gamma (IFN-γ) production compared to individual administration. In contrast, anti-RSV G protein mAb (232-1F) that react outside the central conserved region was poorly effective in treating RSV disease. The results support the hypothesis that mAbs reacting at or near the central conserved region of RSV G protein are effective either alone or in combination to prevent or reduce pulmonary disease associated with RSV infection.

## Methods

### Ethics Statement

The study was performed in accordance with the Guide for the Care and Use of Laboratory Animals of the National Institutes of Health. The protocol was approved by the Centers for Disease Control and Prevention (CDC) Institutional Animal Care and Use Committee (Protocol Number: 1771HAYMOUC).

The human samples used in this study were obtained through a contract between the CDC and Emory University Transfusion Services. Ethics approval for the sample collection and use was approved by the CDC (Protocol Number: 1652) and Emory University (Protocol Number: 00045947) Institutional Review Boards. The donor identity is held only by Emory University Transfusion Services and was not released to CDC in accordance with both institutions approved IRB protocols. The samples used in this study were de-identified and analyzed anonymously.

### Animals

Six-to-eight week old, specific-pathogen-free, female BALB/c (The Jackson Laboratories) mice were used in all experiments. The mice were housed in microisolator cages and fed ad libitum.

### Virus Infection and Tissue Collection

The A2 strain of RSV was used in all experiments and propagated in Vero cells (ATCC CCL 881) as previously described [14]. Mice were anesthetized by intraperitoneally (i.p.) administration of Avertin (2% 2,2,2-tribromoethanol, 2% tert-amyl-alcohol, 180–250 mg/kg), and intranasally (i.n.) challenged with 10^6^ plaques forming units (PFU) of RSV in serum free DMEM in a 50 µL volume. At days 3, 5, 7 and 11 post-challenge, mice were anesthetized with Avertin and exsanguinated by severing the right auxiliary artery. Bronchoalveolar leukocyte (BAL) cells were collected by lavaging the lungs three times with 1ml sterile Dulbecco’s PBS. Lungs were harvested and stored at −80°C until use. No fewer than three mice per treatment per time point were examined.

### Monoclonal Antibody Treatment

On day 3 after infection, mice were i.p. treated with 300 µg/mouse anti-RSV G mAb 130-6D, mAb 232-1F [24], or 300 µg/mouse control normal mouse immunoglobulin G (IgG) (Thermo Fisher Scientific), or for combination experiments, mAbs were administered either alone or in combination at suboptimal doses of 75 µg for mAb 131-2G, 150 µg for mAb 130-6D or 75–150 µg for control IgG. The suboptimal doses of the mAbs were determined based on previous in vivo data (data not shown). Endotoxin concentrations in the mAbs were less than 5 endotoxin units per mg of antibody. mAb 131-2G, mAb-130-6D and mAb 232-1F do not have in vitro neutralizing activity [24].

### Flow Cytometry

The procedure used for extracellular staining of BAL cells was modified for microculture staining as previously described [14]. Briefly, BAL cells were blocked in 10% normal mouse serum in staining buffer (D-PBS with 1% bovine serum albumin) and then stained with the appropriate combinations of fluorescein isothiocyanate-, allophycocyanin-, or phycoerythrin-labeled anti-CD4, anti-CD8, anti-CD3ε, anti-CD45R/B220, anti-CD11b, anti-PMN cell (RB6-8C5), anti-NK cell (DX5), and mouse isotype antibody controls (eBioscience). The distribution of cell surface markers was analyzed on a BD LSRII flow cytometer using FACSDiva software (BD Biosciences) from 10,000 lymphocyte-gated events.

### Chemotaxis Assay

Chemotaxis assays were performed using a modified Boyden chamber with an 8 micron pore size filter as previously described [20]. Human peripheral blood mononuclear cells (PBMC) were freshly isolated by Ficoll gradient from three random donors, pooled and stored in freezing medium (10% DMSO, 12.5% human serum albumin (HSA, Gemini Bio-Products), 77.5% fetal bovine serum (FBS, Life Technologies) at −152°C until use. PBMCs were rested in 1% HSA in RPMI up to 24hrs hours prior to assay. The lower chamber contained purified mAb anti-RSV-G (131-2G), (130-6D), 232-1F or anti-RSV-F (131-2A) antibody with RSV A2-G protein, at the concentrations indicated, relative to purified RSV strain A2 G protein (22.5 µg/mL). PBMCs in chemoattractant medium (serum-free RPMI +0.1% BSA), were loaded in the upper chamber and incubated at 37C for 12–14 hrs. The PBMCs that migrated to the lower chamber were counted and the percentage of inhibition of chemotaxis was determined by the following formula: 100×[1-(total number of cells that migrated toward RSV G protein in the presence of antibodies)/(total number of cells that migrated toward RSV G protein in the absence of antibodies)].

### IFN-γ and IL-4 Enzyme Immunoassays (ELISAs)

IL-4 and IFN-γ levels in cell-free bronchoalveolar lavage (BAL) fluid were detected using a capture ELISA in accordance with the manufacturer’s instructions (eBioscience). All samples were run in duplicate and the assays repeated three independent times.

### Virus Titers

Viral titers in the lungs of RSV-infected mice were determined as previously described [26]. Briefly, lungs were weighed, homogenized in 1 mL/lung of sterile Dulbucco PBS +25% sucrose, and 10-fold serial dilutions (in serum-free DMEM) of the lung homogenates were added to confluent Vero cell monolayers in 24-well plates. After adsorption for 2 h at 37°C, cells monolayers were overlaid with tissue culture DMEM (Life Technologies) containing 10% fetal bovine serum, incubated at 37°C for 3 to 5 days, and then enumerated by immunostaining with mAbs against the G and F proteins (232-1F, 130-6D, and 131-2A, respectively).

### Real-Time Quantitative Reverse-transcriptase PCR (Real-time qRT-PCR)

Total RNA was extracted from homogenized lung tissue using a RNA purification kit (QIAGEN) per manufacturer’s protocol and stored at −80°C until use. Expression of the RSV matrix (M) gene (forward, position 3257-3282) 5′-GGC AAA TAT GGA AAC ATA GCT GAA-3′ and (reverse, position 3312-3340) 5′-TCT TTT TCT AGG ACA TTG TAY TGA ACA G-3′) was determined by real-time quantitative RT-PCR performed on a Stratagene3000 detection system (Agilent Technologies). Threshold cycles (*C*t) for each sample were calculated and serial dilutions of known PFUs of RSV RNA were used to obtain a standard curve.

## Results

### Therapeutic Treatment with an Anti-RSV G Monoclonal Antibody Reactive to G Protein Central Conserved Region Reduces Pulmonary Inflammation

**Figure 1:**
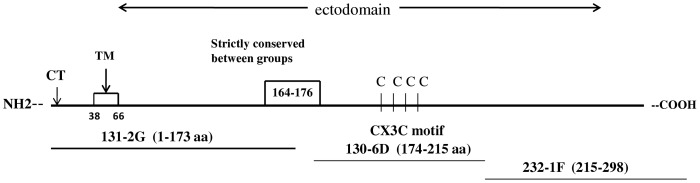
Linear representation of anti-G monoclonal antibody regions of reactivity across the RSV G glycoprotein. The cysteine noose region in the G protein is indicated as C, while the transmembrane and cytoplasmic domains are indicated by TM and CT, respectively. mAb 131-2G recognizes an epitope proximal to the central conserved region at a.a 1–173. mAb 130-6D recognizes an epitope within the central conserved region at a.a 174–215. mAb 232-1F reacts to an epitope outside the central conserved region at a.a. 215–298.

**Figure 2:**
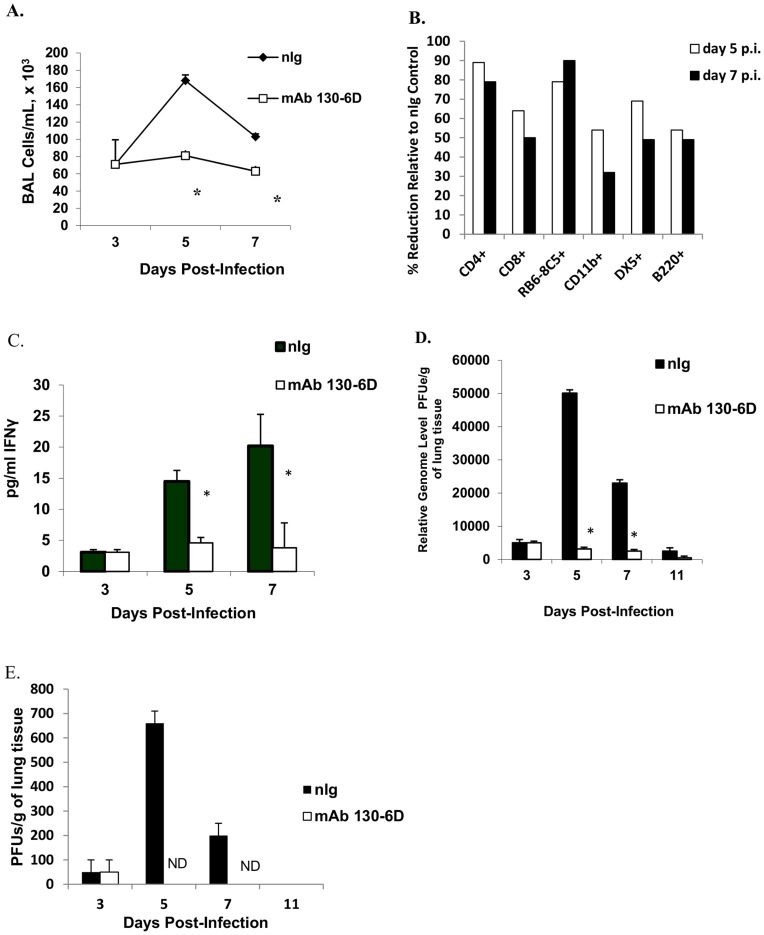
Effects of mAb 130-6D treatment on pulmonary leukocyte trafficking and RSV titers. (A) The BAL cells from normal Ig antibody control (nIg, 300 µg/mouse) and anti-G mAb (mAb 130-6D; 300 µg/mouse) treated mice were harvested on days 3, 5, and day 7 p.i. The data are expressed as the mean number of BAL cells per ml (±standard error, SE). (B) Percent reduction in total CD4^+^, CD8^+^, RB6-8C5^+^, CD11b^+^, DX5^+^ and B220^+^ cell types after mAb 130-6D treatment relative to total cells type after nIg treatment at days 5 and 7 p.i. (C) The levels of IFN-γ cytokine production in BAL cell-free supernatant are expressed in pg/ml. (D) The level of RSV replication in lung tissue was evaluated by real-time RT-PCR (qRT-PCR) for M gene expression (relative genome level equivalent PFU/g of lung tissue x10^3^) and infectious virus titers were determined by immunostaining plaque assay (PFU/g of lung tissue) (E). Asterisks indicate a significant difference (p<0.05) between nIg-treated and antibody treated mice. Results are representative of three independent experiments. ND indicates virus titers below the level of detection.

mAb 130-6D recognizes an epitope on the RSV A2 G protein between amino acid (aa) 174 and 215, a site that overlaps with the central conserved region of RSV G protein [24], [25] (Figure 1). To determine the therapeutic effectiveness of mAb 130-6D, BALB/c mice were i.p. treated with an optimal dose of antibody (300 µg) at day 3, post-RSV infection. Mice treated with mAb prior to the peak of virus replication and the peak inflammatory response to RSV infection [21] had a 75% reduction (p<0.05) in total BAL cells compared to mice similarly treated with normal IgG-treated (Figure 2A). This decrease in cell number was associated with a decrease in most cell types in the BAL with a marked reduction for RB6-8C5^+^ PMNs (79–90%), CD4^+^ cells (79–89%), CD8^+^ cells (50–64%), and DX5^+^NK cells (49–69%) at days 5 and 7 p.i (Figure 2B) and continued through day 11 p.i. (data not shown). Consistent with decreased cell infiltration, the levels of the Th1-type cytokine, interferon gamma (IFN-γ), but not IL-4 levels (data not shown) in the cell-free BAL supernatant were markedly decreased after mAb 130-6D treatment (Figure 2C). Early treatment with mAb 130-6D significantly (p<0.05) inhibited RSV viral gene expression (Figure 2D) and replication (Figure 2E) at all time points examined compared to control antibody treatment. Taken together these results show that early treatment with anti-RSV G mAb 130-6D inhibits major aspects of the pulmonary inflammatory response, a feature similar to previous findings using anti-RSV G mAb 131-2G [21], and support the hypothesis that anti-G antibodies that react at or proximal to the central conserved region can block RSV G protein activities in vivo.

### Therapeutic Treatment with an Anti-G mAb (232-1F) Reacting Outside the G Protein Central Conserved Region does not Protect from RSV Disease

**Figure 3:**
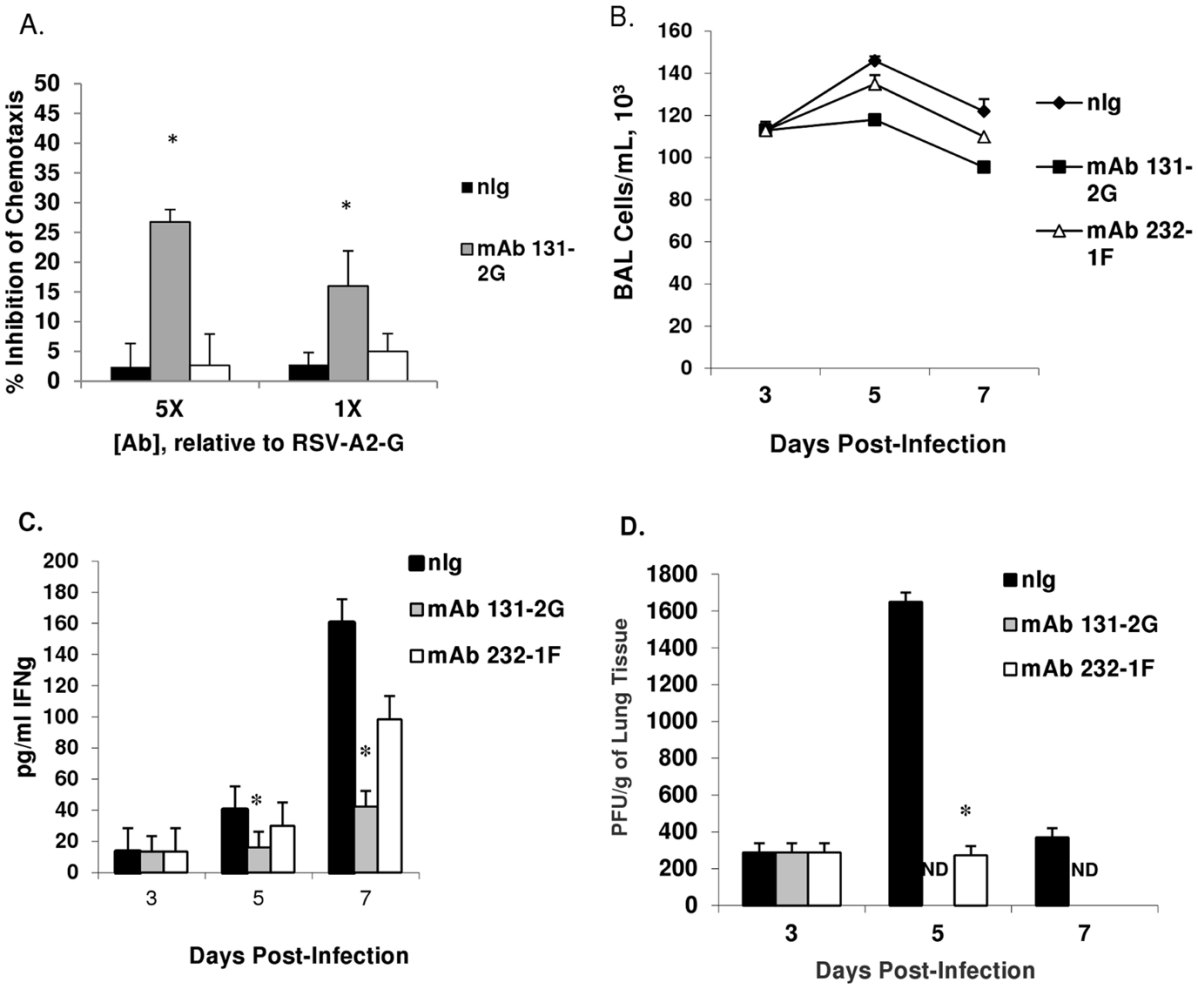
mAb 232-1F does not block RSV G protein induced leukocyte migration or RSV-associated pulmonary inflammation. (A) mAb 232-1F was examined for its ability to block RSV G protein induced leukocyte migration in vitro compared to mAb 232-1F and mAb 131-2G. An anti-RSV F antibody (nIg) was used a negative control. Data is expressed as percent inhibition of leukocyte chemotaxis. (B) BAL cells from antibody-treated (300 µg/mouse, nIg, mAb 232-1F and mAb 131-2G) mice were harvested on days 3, 5 and day 7 post-RSV infection. The data are expressed as the mean number of BAL cells (±SE) per ml. (C) IFN-γ cytokine production (pg/mL ±SE) in cell-free BAL fluid were determined and the virus titers were determined by immunostaining plaque assay (PFU/g of tissue; ±SE) (D). Asterisks represents statistical significance (p<0.05) as determined by comparing nIg treated and mAb treated group. Representative results from two separate experiments are shown. ND indicates virus titers below the level of detection.

Monoclonal antibody 232-1F recognizes an epitope on RSV A2 G protein between a.a 215 and 298, a region that is discrete from the central conserved motif (Figure 1) [24], [25]. In vitro, mAb 232-1F did not neutralize RSV [26], had limited ability to inhibit RSV G protein binding to CX3CR1-transfected 293 cells (data not shown) and did not significantly prevent RSV G protein induced leukocyte migration (<5% inhibition), compared to mAb 131-2G (15 to 27% inhibition) (Figure 3A). The inability of mAb 232-1F to block RSV G protein induced leukocyte migration was comparable to anti-RSV F protein mAb 131-2A, a negative control mAb for inhibition of RSV G protein-mediated lymphocyte migration.

When administered early after RSV infection (day 3 p.i.), mAb 232-1F was less effective at reducing pulmonary inflammation after RSV infection. Compared to mAb131-2G, treatment with 232-1F was markedly less effective at decreasing CD4^+^, DX5^+^, CD8^+^, CD11b^+^, and PMN (RB6-8C5^+^) cells at days 5 and 7 p.i. (Table 1 and Figure 3B). There was no significant difference in BAL cell numbers following treatment with mAb 232-1F compared to nIg controls (Figure 3B); however, cell numbers were significantly (p<0.05) reduced after mAb 131-2G treatment compared to nIg controls consistent with previous findings [21]. Further, the levels of IFN-γ in the cell-free BAL supernatants of mAb 232-1F treated mice were not significantly decreased relative to levels from mice treated with nIg or with mAb 131-2G on day 7 p.i. (Figure 3C). Interestingly, the titer of infectious virus (Figure 3D) was significantly decreased after mAb 232-1F compared to control treated mice but to a lesser extent than that associated with mAbs 131-2G or 130-6D (data not shown). On day 5 p.i., mAb 232-1F treated mice had detectable virus but 5-fold less than those treated with nIg while mAb 131-2G-treated mice had no detectable virus. Differences in cell numbers and IFN-γ expression between treatment with mAb 232-1F and mAb 130-6D were similar to those seen with mAb131-2G noted above (data not shown).

**Table 1:** Pulmonary leukocyte trafficking after anti-RSV G protein mAb 232-1F treatment compared to mAb 131-2G treatment after RSV infection^a^.

Day p.i	Phenotype^a^	nIg (×10^3^)	mAb 131-2G (×10^3)^	% Reduction^b^	mAb 232-1F (×10^3^)	% Reduction^b^
**5**	CD4	25.3±1.4	8.6±1.2	**66** d	23.1±1.0	1
	B220	15.0±1.0	6.4±0.5	**57** c	8.8±0.4	**41** c
	NK	18.9±1.0	9.2±0.6	**52** c	15.4±0.7	18
	CD8	17.5±1.0	4.6±0.6	**74** d	15.6±0.7	10
	CD11b	68.6±3.8	30.1±3.5	**56** c	60.6±2.6	12
	PMN	15.2±1.0	4.9±0.5	**67** d	25.2±1.1	0
**7**	CD4	21.8±1.0	8.5±0.5	**61** d	12.9±0.7	**40** c
	B220	14.6±0.6	8.1±0.4	**45** c	9.1±0.5	**38** c
	NK	18.5±0.8	8.7±0.4	**52** c	11.8±0.6	**37** c
	CD8	30.4±1.2	9.5±1.0	**69** d	24.0±1.3	21
	CD11b	74.7±3.0	45.4±2.4	39	66.0±3.4	12
	PMN	12.8±0.5	1.1±0.5	**91** d	12.4±0.1	0

a RSV A2-infected mice were treated with normal immunoglobulin G (nIg) or anti-RSV G mAb 232-1F or mAb 131-2G on day 3 after infection. Bronchoalveolar lavage (BAL) samples from 4 mice per group were examined on days 3, 5, 7 and 11 after infection. Representative data from days 5 and 7 are shown.

Data are the mean total number of BAL cells expressing CD4, CD8, B220 (B cells), NK (natural killer cells), CD11b (macrophages and/or monocytes), or RB6-8C5 (neutrophils [PMN]) per lung on day 5 and 7 after infection. Standard errors of the mean are also shown.

b Percent reduction is the change in total cell type after anti-RSV G mAb treatment relative to the total cell type after treatment with IgG.

c *p*<0.05 between nIg and mAbs treated mice.

d *p*<0.001 between nIg and mAbs treated mice.

### Anti-RSV G Protein Monoclonal Antibodies used in Combination Significantly Reduce RSV G Protein Mediated Lymphocyte Chemotaxis in vitro

**Figure 4:**
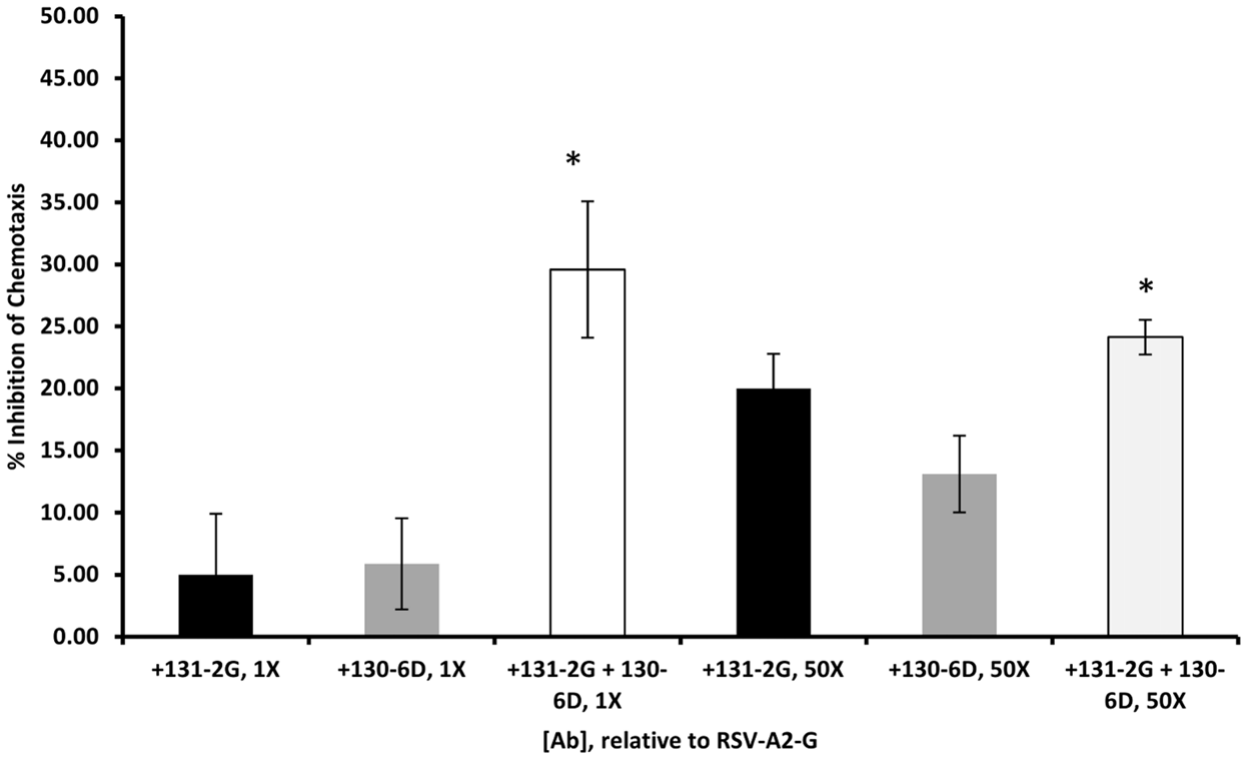
Antibody inhibition of leukocyte migration by mAbs 130-6D and 131-2G. The inhibition of RSV A2 G protein – mediated leukocyte chemotaxis by anti-RSV G antibodies (130-6D and 131-2G) was determined as described in Materials and Methods. Antibody was included in the lower chamber of the chemotaxis chamber, along with purified RSV A2 G protein, at an equal concentration (1X) (22.5 µg/mL) to that of purified RSV G protein (22.5 µg/mL) up to a 50-fold concentration (50X) of mAb to purified RSV G protein. For combination mAb inhibition, 1X and 50X of each antibody were used. Each condition was assayed three times per experiment. Data is presented as an average percent inhibition of RSV A2 G protein mediated leukocyte chemotaxis ± standard deviation relative to the amount of chemotaxis induced by RSV A2 G protein alone. Asterisk indicates significant difference in inhibition (p<0.05) as determined by comparing to 131-2G (1X) or 130-6D (1X) mAb inhibition alone to combination of 131-2G and 130-6D mAbs (1X) or 130-6D (50X) mAb inhibition alone to combination of 131-2G and 130-6D mAbs (50X).

**Figure 5:**
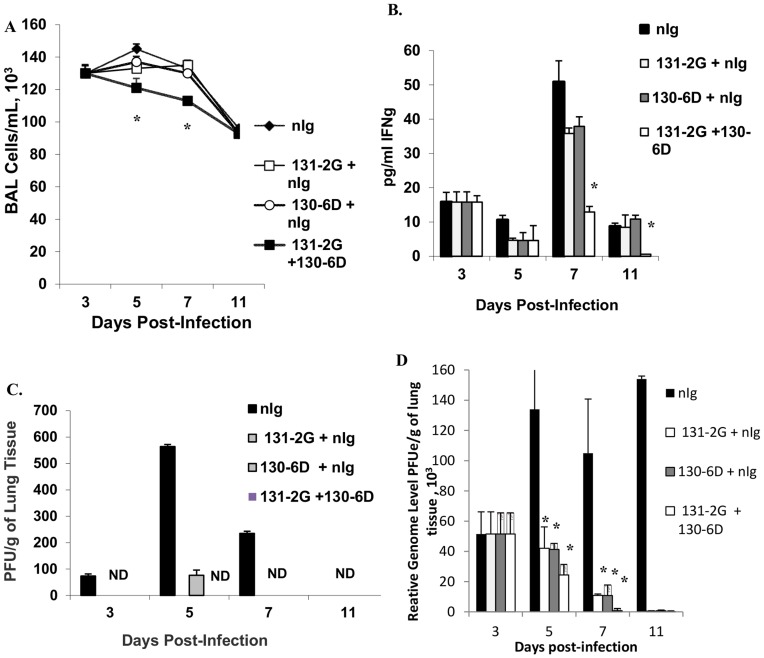
Marked reduction in RSV-associated pulmonary inflammation after early anti-RSV G mAbs combination treatment. RSV-infected mice were treated with suboptimal doses of either anti-G mAb (130-6D, 150 µg/mouse) and normal Ig antibody control (nIg, 75 µg/mouse), anti-G mAb (131-2G, 75 µg/mouse) and normal Ig antibody control (nIg, 75 µg/mouse), normal Ig antibody control (nIg, 225 µg/mouse) alone or both anti-G mAbs in combination (75 µg 130-6D +150 µg 131-2G/mouse). BAL cells were harvested on days 3, 5, 7 and 11 p.i. Shown are total bronchoalveolar lavage (BAL) cell numbers (±SE) per ml (A), (B) interferon-γ (IFN-γ) (pg/ml ±SE) cytokine levels in cell-free BAL lavage fluid. (C) Virus titers (PFU/g of tissue; ± SE) and M gene expression (relative genome level equivalent PFUe/mL lung tissue ± SE) (D) in the lungs of RSV-infected mice were determined. Results are representative of three independent experiments with no fewer than three mice per time point. Asterisks indicate a significant difference (p<0.05) between nIg-treated mice and antibody-treated mice. ND indicates virus titers below the level of detection.

Consistent with previous chemotaxis results using mAb 131-2G, the mAb 130-6D inhibited RSVA2 G protein mediated lymphocyte chemotaxis in a dose-dependent manner with optimal inhibition (20–25%) reached at an antibody concentration 50 fold higher than RSV A2 G protein (data not shown). At a concentration similar to that for RSV G protein (22.5 µg/ml, 1X), neither antibody significantly affected lymphocyte migration, inhibiting between 5 to 15% of lymphocyte chemotaxis. However, when the mAbs 131-2G and 130-6D (1X) were used in combination, inhibition of RSV G protein induced lymphocyte migration was significantly increased compared to inhibition by either antibody alone (25 to 34% together, 5–8% individually, p<0.002) (Figure 4). At a higher antibody concentrations, the addition of mAb 131-2G to mAb 130-6D significantly (p<0.05) increased the inhibition of lymphocyte migration as compared to mAb 130-6D alone. The combination of mAbs used at a lower concentration was more effective at inhibiting RSV G protein induce lymphocyte chemotaxis than each antibody alone at a 50-fold higher concentrations (Figure 4). These findings suggest that a combination of anti-RSV G antibodies that block RSV G protein induced chemotaxis in vitro may be more effective in vivo to decrease RSV-associated pulmonary inflammation in vivo. To specifically address this possibility, a combination of mAb 131-2G (75 µg/mouse) and 130-6D (150 µg/mouse) at sub-optimal concentrations were administered to mice at day 3 post-RSV infection. At sub-optimal concentrations, neither mAb 130-6D nor 131-2G alone significantly reduced pulmonary cell infiltration or pro-inflammatory cytokine production following RSV infection (Figure 5A and 5B).

However, treatment of both mAbs in combination resulted in a significant reduction (p<0.01) in pulmonary leukocyte trafficking at days 5 and 7 p.i. (Figure 5A) with a marked reduction for RB6-8C5^+^ PMNs (66%), CD4^+^, CD8^+^ cells (74% and 53% reduction respectively), and a modest reduction for DX5^+^ NK cells and B220^+^ (36% and 47% reduction, respectively) by day 5 p.i. (Table 2). Further, the level of IFN-γ in BAL fluids of mice treated with the combination of anti-G mAbs was significantly reduced by 75 to 87% (p = 0.002) compared to nIg-treated mice as early as day 5 p.i. (Figure 5B). A similar decrease in the already low IL-4 levels was observed in mice given the combination mAb treatment (p<0.01) (data not shown). Interestingly, by days 5 and 7 p.i., the viral load was reduced to below the limit of assay detection after either treatment with mAb 131-2G alone or mAb 130-6D and mAb 131-2G in combination (Figure 5C). In contrast to infectious virus, and as previously demonstrated by real-time PCR [25], RSV M gene expression was detected in all RSV-infected, nIg or mAb-treated mice at all time points examined however the message levels were three to five-fold lower (p<0.05) in mAb-treated mice compared to nIg-treated mice (Figure 5D).

**Table 2:** Pulmonary Leukocyte Trafficking after Treatment of a Combination of Anti-Respiratory Syncytial Virus (RSV) G Protein Monoclonal Antibodies (mAbs) 131-2G and 130-6D on Day 3.

Days p.i	Phenotype^a^	nIg (10^3^)	131-2G (10^3^)	% Reduction^b^	130-6D (10^3^)	% Reduction^b^	130-6D +131-2G	% Reduction^b^
***5***	CD8	16.5±1.0	16.6±2.5	0	22.1±1.0	0	7.7±0.4	**53** c
	CD4	27.5±1.5	23.2±1.4	15	13.5±0.6	**51** c	6.9±0.4	**74** d
	B220	16.5±0.9	9.3±0.6	**43** c	4.3±0.2	**73** d	8.7±0.8	**47** c
	NK	20.6±1.1	15.9±1.0	22	31.7±1.3	**0**	13.0±0.8	36
	CD11b	10.6±0.6	78.9±4.9	25	67.7±2.9	36	63.7±0.4	40
	PMN	25.7±0.1	16.9±1.0	34	16.8±0.7	35	8.7±0.5	**66** d
***7***	CD8	50.0±1.8	38.5±1.5	23	51.8±2.5	0	29.7±0.2	40
	CD4	24.9±0.9	18.4±0.7	26	13.2±0.6	**47** c	10.5±0.2	**57** c
	B220	14.1±0.5	8.8±0.3	38	11.1±0.5	21	8.3±1.4	41
	NK	32.8±1.2	20.6±0.9	37	12.9±0.6	**60** c	13.8±0.5	**60** c
	CD11b	98.8±3.6	77.8±3.2	21	75.8±3.6	23	58.4±0.8	41
	PMN	20.3±0.7	11.3±0.5	**44** c	11.1±0.5	**45** c	4.8±1.9	**76** d
***11***	CD8	35.2±2.2	27.4±1.8	22	37.5±2.4	0	23.6±2.1	33
	CD4	14.6±1.0	9.5±0.7	35	14.0±1.0	4	9.8±2.1	32
	B220	9.9±0.6	7.6±0.5	24	9.3±0.6	6	8.2±1.0	18
	NK	8.4±0.5	8.4±0.5	1	8.3±0.5	2	5.8±0.8	31
	CD11b	49.2±3.1	50.0±3.2	0	57.6±3.6	0	39.8±0.5	19
	PMN	11.2±0.7	13.2±0.9	0	14.1±0.9	0	8.3±0.6	25

a RSV A2-infected mice were treated with suboptimal doses of normal immunoglobulin G (nIg) or anti-RSV G mAbs (130-6D and 131-2G) either alone or in combination on day 3 after infection. Bronchoalveolar lavage (BAL) samples from 4 mice per group were examined on day, 3, 5, 7 and 11 after infection. Representative data from days 5, 7 and 11 are shown. Data are the mean total no. of BAL cells expressing CD4, CD8, B220 (B cells), NK (natural killer cells), CD11b (macrophages and/or monocytes), or RB6-8C5 (neutrophils [PMN]) per lung on day 5, 7 and 11 after infection. Standard errors of the mean are also shown.

b The percent reduction is the change in total cell type after anti-RSV G mAb treatment relative to the total cell type after treatment with IgG.

c *p*<0.05 between nIg and mAbs treated mice.

d *p*<0.001 between nIg and mAb- treated mice.

## Discussion

Over the past decade, numerous studies have focused on the role of RSV G protein and RSV-mediated pathology, and new support for therapeutic targeting of this glycoprotein continues to emerge [21], [22], [23], [28], [29], [30]. Much of the therapeutic focus has been to inhibit RSV G protein misdirection of the host innate immune response. In mouse models, a mAb against the RSV G protein has shown a significant efficacy both in reducing viral load and decreasing the RSV G protein-induced inflammatory response [21], [22], [23], [24], [25], [30]. We have also demonstrated that blocking the RSV G protein CX3C-CX3CR1 interaction with anti-CX3CR1 antibody before RSV challenge of mice vaccinated with formalin-inactivated RSV reduced or eliminated the eosinophilic inflammatory response [11].

In this study, we show that therapeutic treatment with non-neutralizing anti-RSV G mAb (130-6D) that recognizes an epitope within the central conserved region of RSV G protein (174-215 a.a), can prevent RSV G protein induced leukocyte migration and provide protection from inflammatory parameters associated with RSV disease. The anti-inflammatory effect of mAb 130-6D is likely linked in part to its ability to block RSV G protein binding to CX3CR1 through its CX3C chemokine motif [18]. The increase of virus clearance associated with mAb 130-6D treatment, while not expected, was consistent with previous findings using another non-neutralizing anti-G protein mAb, 131-2G. In that study, mAb 131-2G was shown to have distinct functions, i.e. down-regulating aspects of the virus induced host inflammatory response to infection and facilitating virus clearance through an antibody-dependent cell mediate cytotoxicity (ADCC)-dependent mechanism [21], [22]. While there are several possible mechanisms used by non-neutralizing antibodies to mediate increased viral clearance, the mechanism used by mAb 130-6D to mediate virus clearance requires further investigation.

In this study, we also show that therapeutic treatment with mAb 232-1F, a mAb that reacts outside of the central conserved region of RSV G protein (aa 215-298 ) [24], [25], [27] and does not block RSV G protein binding to CX3CR1-transfected 293, did not inhibit RSV G protein-mediated leukocyte chemotaxis, and was less effective in reducing pulmonary inflammation following RSV infection. It is possible that the decrease in inflammation associated with 232-1F was associated with decreased virus replication. Taken together the data presented supports the hypothesis that anti-RSV G mAbs that block the CX3C-CX3CR1 interaction have the ability to inhibit RSV-G mediated activities and protect from immune parameters associated with RSV-infection.

The highest degree of antigenic diversity is found in the RSV G protein and several genetic mechanisms operate in the generation of its diversity [16], [31]. Previous studies have shown that a single anti-RSV G protein mAb can induce escape mutants that can alter antibody binding [32], [33]. Using a combination of anti-RSV G protein mAbs with non-overlapping and non-competing epitopes may ensure better protection against such mAb resistance and enhance therapeutic efficacy. To determine if a combination of anti-RSV G protein mAbs recognizing different RSV G protein epitopes enhanced the ability to block CX3C-CX3CR1 related functions, suboptimal concentrations of mAb 131-2G and mAb 130-6D were tested in combination to protect against RSV infection. The findings showed that the combination of mAbs was associated with greater reduction in pulmonary infiltrate and also increased in vitro measures of RSV-G protein-mediated lymphocytes chemotaxis, and that the effect was greater than either treatment alone. These findings suggest that mAbs could act in a synergistic manner. However, it is not presently clear whether the activity resulting from the combination treatment is additive or synergetic. Our data do raise the possibility that a combination of mAb 131-2G and mAb 130-6D that react at different epitopes may be more effective than either alone.

As the response to natural RSV infection includes antibodies that block RSV G protein CX3C-CX3CR1 interaction and RSV G-mediated leukocyte chemotaxis [22], [23], the results reported here indicate that a combination of anti-RSV G monoclonal antibodies that react at different epitopes on G protein can block G bind RSV G CX3C-CX3CR1 and may improve the effectiveness of immune treatment of RSV infection. These results also suggest that vaccines designed to induce antibodies that react to these sites may help improve efficacy and safety by reducing G protein immune modulation. Importantly, the results also suggest that treatment with antibodies that do not block RSV G CX3C-CX3CR1 interaction will likely be less effective in treating RSV disease, thus this study is important in the development of efficacious RSV vaccine and therapeutic disease intervention strategies.

## References

[1] Panitch HB (2001) Bronchiolitis in infants. Curr Opin Pediatr 13: 256–60. 11389361 10.1097/00008480-200106000-00008

[2] Coffman S (2009) Late preterm infants and risk for RSV. MCN Am J Matern Child Nurs. 34: 378–84. 10.1097/01.NMC.0000363687.27939.e4 19901700

[3] Shay DK, Holman RC, Newman RD, Liu LL, Stout JW, et al (1999) Bronchiolitis-associated hospitalizations among US children, 1980–1996. JAMA 282: 1440–6. 10535434 10.1001/jama.282.15.1440

[4] Hall CB, Iwane MK, Blumkin AK, Edwards KM, Staat MA, et al (2009) The burden of respiratory syncytial virus infection in young children. N Engl J Med 360: 588–98. 19196675 10.1056/NEJMoa0804877 PMC4829966

[5] Murata Y (2009) Respiratory syncytial virus vaccine development. Clin Lab Med 29: 725–39. 19892231 10.1016/j.cll.2009.07.004 PMC2774466

[6] Empey KM, Peebles RS, Kolls JK (2010) Pharmacologic advances in the treatment and prevention of respiratory syncytial virus. Clin Infect Dis 50: 1258–67. 20235830 10.1086/651603 PMC2851489

[7] Win N, Mitchell D, Pugh S, Russell N (1992) Successful therapy with ribavirin of late onset respiratory syncytial virus pneumonitis complicating allogeneic bone transplantation. Clin Lab Haematol 14: 29–30. 1600691

[8] Varga SM, Braciale TJ (2002) RSV-Induced Immunopathology: Dynamic Interplay between the Virus and Host Immune Response. Virology 295: 203–207. 12033778 10.1006/viro.2002.1382

[9] Mejías A, Chávez-Bueno S, Ríos AM, Saavedra-Lozano J, Fonseca Aten M, et al (2004) Anti-respiratory syncytial virus (RSV) neutralizing antibody decreases lung inflammation, airway obstruction, and airway hyperresponsiveness in a murine RSV model. Antimicrob Agents Chemother 48: 1811–22. 15105140 10.1128/AAC.48.5.1811-1822.2004 PMC400529

[10] Krilov LR (2011) Respiratory syncytial virus disease: update on treatment and prevention. Expert Rev Anti Infect Ther 9: 27–32. 21171875 10.1586/eri.10.140

[11] Haynes LM, Jones LP, Barskey A, Anderson LJ, Tripp RA (2003) Enhanced disease and pulmonary eosinophilia associated with formalin-inactivated respiratory syncytial virus vaccination are linked to G glycoprotein CX3C-CX3CR1 interaction and expression of substance P. J Virol. 77: 9831–44. 10.1128/JVI.77.18.9831-9844.2003 PMC224581 12941892

[12] Tripp RA (2004) Pathogenesis of respiratory syncytial virus infection. Viral Immunol 17: 165–81. 15279697 10.1089/0882824041310513

[13] Harcourt J, Alvarez R, Jones LP, Henderson C, Anderson LJ, et al (2006) Respiratory syncytial virus G protein and G protein CX3C motif adversely affect CX3CR1+ T cell responses. J. Immunol 176: 1600–9. 16424189 10.4049/jimmunol.176.3.1600

[14] Tripp RA, Jones L, Anderson LJ (2000) Respiratory syncytial virus G and/or SH glycoproteins modify CC and CXC chemokine mRNA expression in the BALB/c mouse. J Virol 74: 6227–9. 10846112 10.1128/jvi.74.13.6227-6229.2000 PMC112127

[15] Tripp RA, Dakhama A, Jones LP, Barskey A, Gelfand EW, et al (2003) The G Glycoprotein of Respiratory Syncytial Virus Depresses Respiratory Rates through the CX3C Motif and Substance P. J Virol. 77: 6580–6584. 10.1128/JVI.77.11.6580-6584.2003 PMC155004 12743318

[16] Johnson PRJ, Olmsted RA, Prince GA, Murphy BR, Alling DW, et al (1987) Antigenic relatedness between glycoproteins of human respiratory syncytial virus subgroups A and B: evaluation of the contributions of F and G glycoproteins to immunity. J Virol 61: 3163–6. 3305988 10.1128/jvi.61.10.3163-3166.1987 PMC255893

[17] Sullender W (1995) Antigenic analysis of chimeric and truncated G proteins of respiratory syncytial virus. Virology 209: 70–9. 7747486 10.1006/viro.1995.1231

[18] Tripp RA, Jones LP, Haynes LM, Zheng H, Murphy PM, et al (2001) CX3C chemokine mimicry by respiratory syncytial virus G glycoprotein. Nat Immunol 2: 732–8. 11477410 10.1038/90675

[19] Amanatidou VSG, Apostolakis S, Tsilimigaki A, Spandidos DA (2006) T280M variation of the CX3C receptor gene is associated with increased risk for severe respiratory syncytial virus bronchiolitis. Pediatr Infect Dis J 25: 410–15. 16645504 10.1097/01.inf.0000214998.16248.b7

[20] Harcourt JL, Karron RA, Tripp RA (2004) Anti-G protein antibody responses to respiratory syncytial virus infection or vaccination are associated with inhibition of G protein CX3C-CX3CR1 binding and leukocyte chemotaxis. J Infect Dis 190: 1936–40. 15529257 10.1086/425516

[21] Haynes LM, Caidi H, Radu GU, Miao C, Harcourt JL, et al (2009) Therapeutic Monoclonal Antibody Treatment Targeting Respiratory Syncytial Virus (RSV) G Protein Mediates Viral Clearance and Reduces the Pathogenesis of RSV Infection in BALB/c Mice. J Infect Dis 200: 439–47. 19545210 10.1086/600108

[22] Miao C, Radu GU, Caidi H, Tripp RA, Anderson LJ, et al (2009) Treatment with respiratory syncytial virus G glycoprotein monoclonal antibody or F(ab’)2 components mediates reduced pulmonary inflammation in mice. J Gen Virol 200: 1119–23. 10.1099/vir.0.009308-0 PMC2887562 19264600

[23] Radu GU, Caidi H, Miao C, Tripp RA, Anderson LJ, et al (2010) Prophylactic treatment with a G glycoprotein monoclonal antibody reduces pulmonary inflammation in respiratory syncytial virus (RSV)-challenged naive and formalin-inactivated RSV-immunized BALB/c mice. J Virol 84: 9632–6. 20592094 10.1128/JVI.00451-10 PMC2937657

[24] Anderson LJ, Bingham P, Hierholzer C (1988) Neutralization of respiratory syncytial virus by individual and mixtures of F and G protein monoclonal antibodies. J Virol 62: 4232–8. 2459412 10.1128/jvi.62.11.4232-4238.1988 PMC253856

[25] Anderson LJ, Hierholzer JC, Tsou C, Hendry RM, Fernie BF, et al (1985) Antigenic characterization of respiratory sycyntial virus strains with monoclonal antibodies. J Infect Dis 151: 626–633. 2579169 10.1093/infdis/151.4.626

[26] Haynes LM, Anderson LJ, Tripp RA (2002) Neutralizing anti-F glycoprotein and anti-substance P antibody treatment effectively reduces infection and inflammation associated with respiratory syncytial virus infection. J Virol 76: 6873–81. 12072488 10.1128/JVI.76.14.6873-6881.2002 PMC136305

[27] Anderson LJ, Hierholzer JC, Stone Y, Tsou C, Fernie BF (1986) Identification of Epitopes on Respiratory Syncytial Virus Proteins by Competitive Binding Immunoassay. J Clin Virol 23: 475–480. 10.1128/jcm.23.3.475-480.1986 PMC268677 2420819

[28] Kauvar LM, Haynes LM, Tripp RA (2010) Therapeutic targeting of respiratory syncytial virus G-protein. Immunotherapy 2: 655–61. 20874649 10.2217/imt.10.53 PMC3044486

[29] Andersson C, Liljestron P, Ståhl S, Power UF (2000) Protection against respiratory syncytial virus (RSV) elicited in mice by plasmid DNA immunisation encoding a secreted RSV G protein-derived antigen. FEMS Immunol Med Microbiol 29: 247–253. 11118904 10.1111/j.1574-695X.2000.tb01530.x

[30] Collarini EJ, Lee F, Foord O, Park M, Sperinde G, et al (2009) Potent high-affinity antibodies for treatment and prophylaxis of respiratory syncytial virus derived from B cells of infected patients. J Immunol 183: 6338–45. 19841167 10.4049/jimmunol.0901373

[31] Harcourt JL, Karron RA, Tripp RA (2004) Anti-G protein antibody responses to respiratory syncytial virus infection or vaccination are associated with inhibition of G protein CX3C-CX3CR1 binding and leukocyte chemotaxis. J Infect Dis 190: 1936–40. 15529257 10.1086/425516

[32] Langeduk JP, Meloen RH, Taylor G, Furze JM, Oirschot JT (1997) Antigenic structure of the central conserved region of protein G of bovine respiratory syncytial virus. J Virol 71: 4065–4061. 10.1128/jvi.71.5.4055-4061.1997 PMC191558 9094683

[33] Walsh EE, Falsey AR, Sullendar WM (1998) Monoclonal antibody neutralization escape mutants of respiratory syncytial virus with unique alterations in the attachment (G) protein. J Gen Virol 79: 479–487. 9519826 10.1099/0022-1317-79-3-479

